# HIV-1 resistance against dolutegravir fluctuates rapidly alongside erratic treatment adherence: a case report

**DOI:** 10.1016/j.jgar.2022.11.001

**Published:** 2022-11-05

**Authors:** Jeroen J.A. van Kampen, Hanh Thi Pham, Sunbin Yoo, Ronald J. Overmars, Cynthia Lungu, Rizwan Mahmud, Carolina A.M. Schurink, Sander van Boheemen, Rob A. Gruters, Pieter L.A. Fraaij, David M. Burger, Jolanda J.C. Voermans, Casper Rokx, David A.M.C. van de Vijver, Thibault Mesplède

**Affiliations:** a Viroscience department, Erasmus Medical Center, Rotterdam, The Netherlands; b Department of Microbiology and Immunology, McGill University, Canada; c Department of Medical Microbiology and Infectious Diseases, Erasmus Medical Center, Rotterdam, The Netherlands; d Department of Internal Medicine, Erasmus Medical Center, Rotterdam, The Netherlands; e Department of Pediatrics, Subdivision Infectious Diseases and Immunology, Sophia’s Children Hospital, Erasmus Medical Center, Rotterdam, The Netherlands; f Department of Pharmacy, Radboud Institute for Health Sciences, Radboud University Medical Center, Nijmegen, The Netherlands

**Keywords:** Dolutegravir, HIV, Drug resistance, Treatment adherence, Next-generation sequencing, Mutations

## Abstract

**Objectives:**

We report a case of incomplete HIV-1 suppression on a dolutegravir, lamivudine, and abacavir single-tablet regimen with the emergence of the H51Y and G118R integrase resistance mutations.

**Methods:**

Integrase sequencing was performed retrospectively by Sanger and next-generation sequencing. Rates of emergence and decline of resistance mutations were calculated using next-generation sequencing data. Dolutegravir plasma concentrations were measured by ultra-performance liquid chromatography-tandem mass spectrometry. The effects of H51Y and G118R on infectivity, fitness, and susceptibility to dolutegravir were quantified using cell-based assays.

**Results:**

During periods of non-adherence to treatment, mutations were retrospectively documented only by next-generation sequencing. Misdiagnosis by Sanger sequencing was caused by the rapid decline of mutant strains within the retroviral population. This observation was also true for a M184V lamivudine-resistant reverse transcriptase mutation found in association with integrase mutations on single HIV genomes. Resistance rebound upon treatment re-initiation was swift (>8000 copies per day). Next-generation sequencing indicated cumulative adherence to treatment. Compared to WT HIV-1, relative infectivity was 73%, 38%, and 43%; relative fitness was 100%, 35%, and 10% for H51Y, G118R, and H51Y+G118R viruses, respectively. H51Y did not change the susceptibility to dolutegravir, but G188R and H51Y+G118R conferred 7- and 28-fold resistance, respectively.

**Conclusion:**

This case illustrates how poorly-fit drug-resistant viruses wax and wane alongside erratic treatment adherence and are easily misdiagnosed by Sanger sequencing. We recommend next-generation sequencing to improve the clinical management of incomplete virological suppression with dolutegravir.

## 1. Introduction

Antiretroviral drug regimens based on the second-generation integrase strand transfer inhibitors (INSTIs) dolutegravir (DTG) and bictegravir (BIC) are recommended for the initial treatment of HIV infection in the U.S.A. and Europe [1]. A worldwide roll-out of DTG to low- and middle-income countries is in progress [2]. The scarcity of documented resistance mutations after virological failure supports broad DTG and BIC use [3,4]. This so-called ‘high barrier to resistance’ of DTG and BIC creates a knowledge gap about the emergence and maintenance vs. reversion of drug resistance-associated integrase mutations. We report a case of incomplete viral suppression with a single-tablet regimen composed of DTG plus lamivudine (3TC) and abacavir (ABC) and the emergence of an H51Y plus G118R combination of integrase mutations in an antiretroviral treatment-experienced, INSTI-naïve subtype B HIV-1-positive individual. We deemed this case as worth reporting because integrase and reverse transcriptase resistance mutations waxed and waned alongside antiretroviral drug use.

## 2. Materials and methods

### 2.1. Patient consent statement

The patient provided written informed consent for the anonymous publication of their clinical data. People living with HIV who are treated at Erasmus Medical Center are enrolled to the National Database HIV Monitoring Foundation (Special Medical Procedure Act, Article 8 WBMW), a situation that waives the need for institutional ethics approval.

### 2.2. Integrase sequencing and dolutegravir concentrations

Viral RNA purification, Sanger sequencing, and next-generation sequencing (NGS) were performed as previously described [5]. We set the detection limit of genetic variants above 2% and 1000 reads coverage. DTG plasma concentrations were measured as previously published [6]. Based on average C_min_ = 0.8 – 1.2 mg/L and t_1/2_ = 14 hours, DTG concentrations below 0.01 mg/L indicated that DTG had not been taken for four days or longer.

### 2.3. Clonality analysis, viral infectivity, resistance testing, and replication assay

To test the linkage between reverse transcriptase (RT) and integrase mutations, month 36 HIV-1 RNA was reverse transcribed on limit-dilutions and in duplicates (to exclude random recombination) and then cloned and sequenced as described previously [7], except that the following primers were used: 2014F (5’-AGGTACAGTATTAGTAGGAC-3’) and 4727R (5’-AGGGCTTTCATAGTGATGTC-3’). The production of the pNL4.3(H51Y), pNL4.3(G118R), and pNL4.3(H51Y+G118R) viruses; titration; retroviral replication in PM1 cells; and infectivity and drug resistance assays on TZM-bl reporter cells were all performed as previously published [7]. To calculate relative fitness, areas under the replication curves in PM1 cells were normalized to that of the WT virus.

## 3. Results and discussion

### 3.1. Clinical case overview

**Fig. 1.**
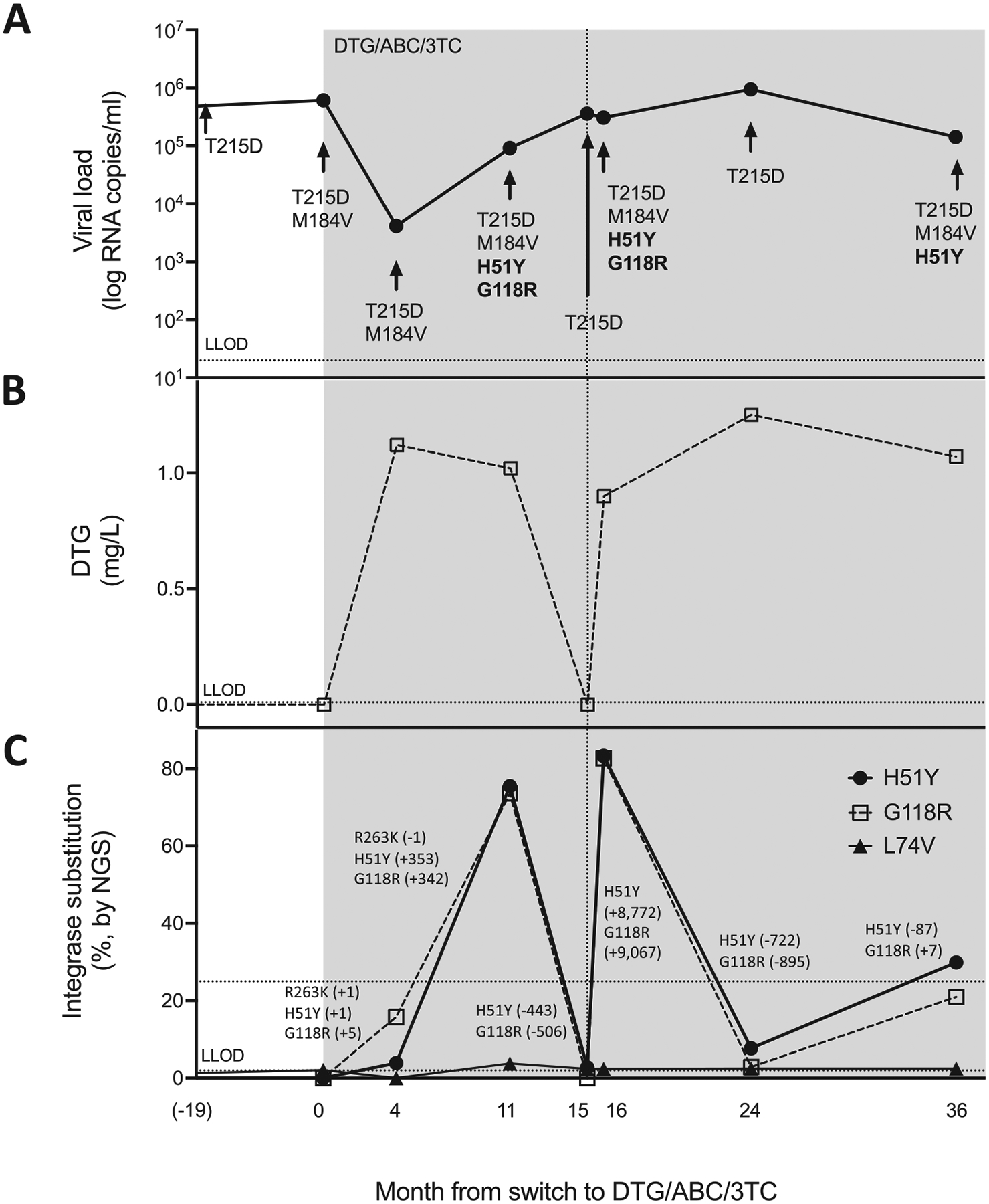
Viral loads, dolutegravir (DTG) concentrations, and genotyping results. Panel A shows the patient’s viral loads from 19 months before to 36 months after switching to a single-tablet regimen of DTG plus abacavir plus lamivudine (DTG/ABC/3TC) (shaded). Resistance mutations detected by Sanger sequencing are indicated. Panel B shows DTG plasma levels. Panel C shows resistance mutations detected by next-generation sequencing (NGS) and linear regression slopes for each interval (in RNA copy/day). Lower limits of detection (LLOD) are shown.

The patient was diagnosed with a subtype B HIV-1 infection in 1993. Treatment was initiated in August 1996 with indinavir, 3TC, and stavudine, and periods of irregular retention in care followed related to issues of illicit substance addiction. In 1999, the patient stopped treatment and resumed in 2002 with ritonavir-boosted lopinavir (r/LPV) plus nevirapine. In 2003, treatment was changed to r/LPV plus 3TC and didanosine (ddI). The patient interrupted treatment from January to May 2004, when the r/LPV+3TC+ddI combination was reinitiated. Treatment was again interrupted at the patient’s initiative from April 2004 to June 2006, and r/LPV+3TC+ddI was restarted. After another patient-initiated interruption from September 2006 to June 2007, the same treatment was again prescribed. In December 2009, treatment was switched to r/LPV plus tenofovir disoproxil fumarate (TDF) and emtricitabine (FTC). From 1999 to 2008, resistance mutations were documented against nucleoside (M41L, T215D/S/Y/A) and non-nucleoside (V108I, V179D) reverse transcriptase (RT) inhibitors by routine Sanger sequencing. No resistance was observed against protease or INSTIs. In August 2013, the CD4+ T-lymphocyte count was 10. In 2015, the patient was switched to a single-tablet regimen of DTG plus lamivudine (3TC) and abacavir (ABC) to facilitate treatment adherence and achieve viral suppression with a CD4+ T-cell count of 10 and a viral load of 6.13E+05 RNA copies/mL of plasma. The patient had not been previously treated with another INSTI. Following this treatment switch, the patient attended blood draws but not appointments with the treating physician. This situation hindered treatment assessment and adjustment. Thirty-six months after switching to DTG/3TC/ABC, the patient was hospitalized with pneumococcal pneumonia, where they were offered various interventions to improve treatment adherence. The CD4+ T-cell count was 100, and the plasma viral load was 1.42E+05 copies/mL. A genotypic resistance test using Sanger sequencing revealed the H51Y integrase mutation. Since H51Y by itself has a minimal effect on susceptibility to DTG [8], we suspected the presence of additional mutations. We thus performed retrospective genotypic drug resistance testing by Sanger sequencing and NGS and DTG drug level quantification on all existing samples (Fig. 1). It should be noted that, at our hospital, Sanger sequencing is not performed on all samples with detectable viral loads; instead, it is done on-demand.

### 3.2. Sanger and NGS sequencing results

The viral load declined during the first four months after DTG/3TC/ABC initiation from 6.13E+05 to 4.13E+03 RNA copies/mL of plasma, and DTG plasma levels were adequate, excluding major drug absorption issues (Fig. 1). No integrase mutation was observed by Sanger sequencing. In contrast, NGS uncovered the H51Y (4%), G118R (16%), and R263K (4%) DTG-signature substitutions [4], which developed at a linear rate of 1 to 5 RNA copies/day. Between months 4 and 11, H51Y and G118R expansion accelerated to ~350 RNA copies/day, and they became detectable by Sanger sequencing despite proper DTG plasma levels. R263K was lost. The CD4+ T-cell count at month 11 was 110 and the viral load had increased to 9.14E+04 RNA copies/mL. No integrase mutation was found by Sanger sequencing at month 15, which yielded no quantifiable DTG in plasma and an increasing viral load (3.61E+05 RNA copies/mL). Only H51Y (2.8%) was detectable by NGS. During this period of non-adherence, H51Y and G118R receded by ~450 RNA copies/day. On month 16 (VL=3.07E+05 RNA copies/ml), H51Y and G118R were both detected by Sanger sequencing, and they dominated (~80%) the retroviral population by NGS, concomitantly with sufficient DTG plasma concentrations (0.90 mg/L). The rapid resumption of the two integrase resistance mutations averaged ~9,000 RNA copies/day. After 24 months, viral loads continued to rise to 9.49E+05 RNA copies/mL of plasma, and H51Y and G118R were no longer detected by Sanger sequencing despite a DTG plasma concentration of 1.2 mg/L. H51Y and G118R declined by ~800 RNA copies/day but persisted below the threshold of detection by Sanger sequencing. At month 36 (VL = 1.42E+05 RNA copies/mL), H51Y was detected by Sanger sequencing and NGS, whereas G118R (21%) was only visible by NGS.

Like the integrase mutations, the 3TC-resistant M184V RT substitution was not detected by Sanger sequencing at months 15 and 24. In contrast, the thymidine analog mutation (TAM) revertant T215D was consistently detected by Sanger sequencing throughout. The minor integrase polymorphism L74V was also consistently detected at months 15 and 24 by NGS. These two observations argued against the possibility of sampling or processing errors.

### 3.3. Molecular virology

**Fig. 2.**
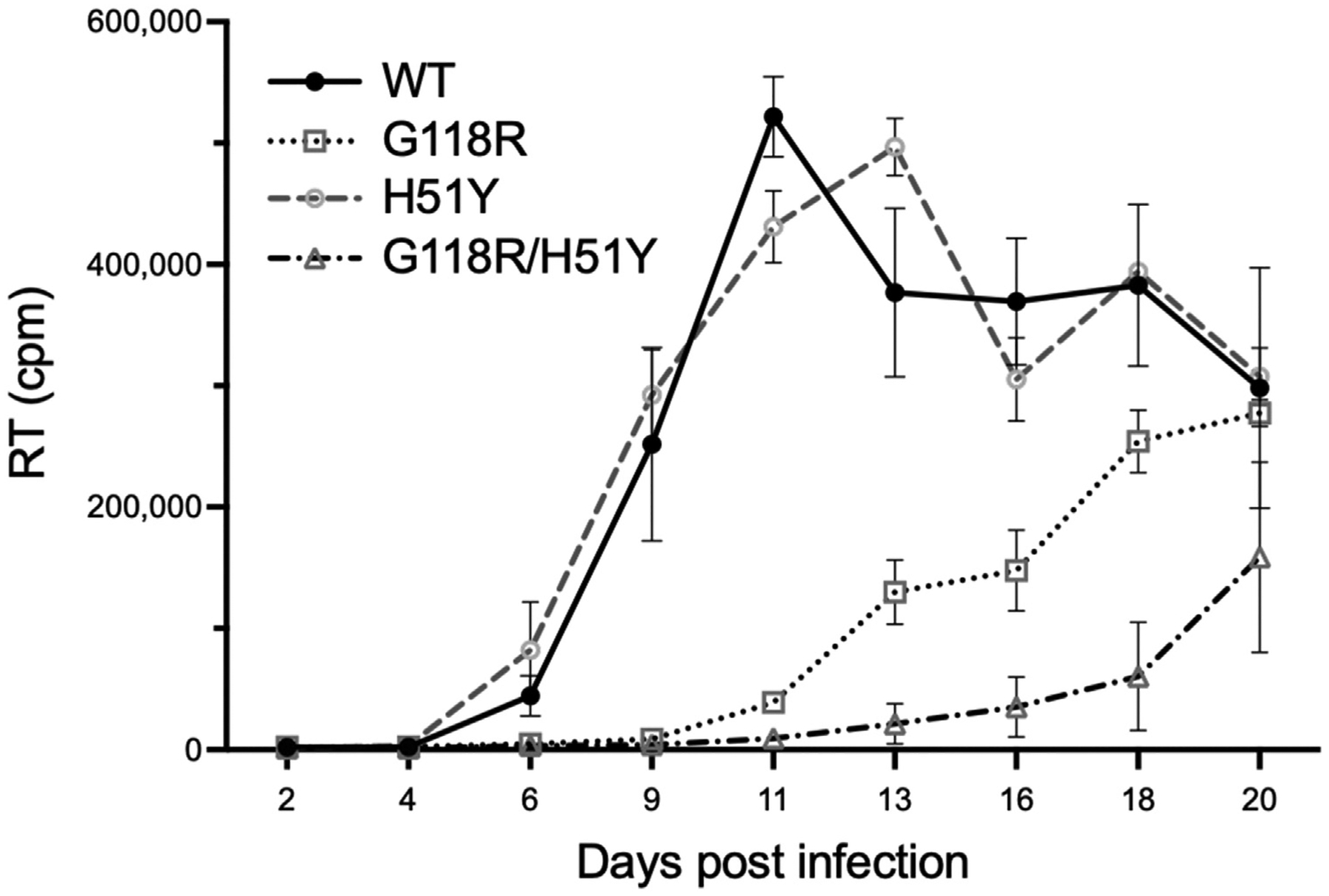
Replication of WT, H51Y, G118R, and H51Y+G118R HIV-1. Retroviral replication over time measured by reverse transcriptase (RT) activity in cell culture fluids (count per minute, cpm). The HIV-1 genotype at the time of infection is indicated.

Clonal analysis of retroviruses circulating at month 36 showed that M184V and integrase mutations co-existed on single viral genomes in 42% of viruses (not shown). Cell-based assays (Table 1, Fig. 2) confirmed previous reports that H51Y by itself has minor effects on infectiousness, replicative capacity, and resistance against DTG [8]. Both G118R and H51Y+G118R viruses displayed reduced infectiousness (38% and 43% of WT, respectively). The H51Y+G118R virus performed less well in replication assays than G118R (relative fitness = 10% vs. 30%, respectively). G118R and H51Y+G118R reduced susceptibility to DTG by 7.2- and 28.3-fold, respectively.

### 3.4. Discussion

The characterization of the effects of H51Y and G118R on infectivity, fitness, and susceptibility to DTG allowed us to interpret our clinical observations as follows: impaired replicative capacity contributed to the waning of drug-resistance mutations during episodes of non-adherence to treatment, whereas resistance levels helped mutations to rematerialize quickly after drug resumption. Our case report exemplifies how the decline of integrase resistance mutations during a period of non-adherence to treatment can prevent their diagnosis by Sanger sequencing. Using linear regression, we calculated that G118R fell under the Sanger sequencing limit of detection (20% of the retroviral population) in 42 days despite a concomitant increase in viral loads. This rapid decline happened despite the severe immunodeficiency of the patient who consistently had CD4+ T-lymphocyte counts <110. We speculate that immune competence (i.e. higher CD4+ T-cell counts) can accelerate the disappearance of integrase resistance mutations. The average rate of decline of integrase mutations (−641 [IC_95_ = 312−971] RNA copies/day) over two periods of non-adherence to treatment (months 11–15 and 16–24) was comparable or lower to those reported for R263K in two separate clinical cases with BIC and DTG (~320 and ~3,000 RNA copies/day, respectively) [9,10]. The current recommendation to perform resistance genotyping within four weeks of virological non-suppression may not be sufficient in the context of typical low-level viremia during failure with DTG. Instead, we recommend that resistance genotyping be performed by NGS, ideally on the earliest available positive sample.

The DTG/3TC/ABC single-tablet regimen and linkage of resistance mutations against DTG and 3TC on single HIV-1 genomes explain why M184V paralleled the decline and resurgence of integrase mutations. Likely, clinical and virological factors, including viral loads, adherence to treatment, viral fitness, immune competence (i.e. CD4+ T-cell counts), and drug resistance, all contribute to the pace of changes in the retroviral population. Drug adherence can be particularly challenging to evaluate, as adequate drug plasma levels at scheduled visits can result from ‘white coat adherence’ before blood collection. Methods to evaluate cumulative treatment adherence, such as unscheduled drug level dosing or antiretroviral drug quantification in hair, can be intrusive, negatively affect linkage to care, necessitate additional consent, or be methodologically challenging to implement [11]. We found that monitoring drug-resistant viruses by NGS could serve to indicate cumulative treatment adherence. This is best illuminated by the decline in drug-resistant viruses between months 16 and 24 that indicated cumulative non-adherence despite detectable levels of DTG at month 24. NGS was superior to merely monitoring viral loads because drug-resistant virus copy numbers changed faster than the viral load, as illustrated by the period between months 15 and 16 when viral loads did not significantly change, whereas H51Y and G118R rematerialized quickly. In addition, NGS may be easier to implement than other methods aimed at evaluating cumulative drug adherence because it does not necessitate additional consent.

There is a general understanding that DTG- and BIC-based treatment regimens are highly robust against virological failure and the development of resistance mutations. This notion is supported by several studies, including the NADIA clinical trial that compared DTG with the protease inhibitor darunavir and demonstrated high levels of suppression regardless of nucleotide/nucleoside reverse transcriptase inhibitor resistance [12–14]. However, neither DTG nor BIC are completely impervious to the issue of resistance [4,9,10,15–17]. In addition, this notion of robustness against resistance may delay the decision to perform sequencing when failure occurs. Our report accidentally illustrates how such delays may prevent the diagnosis of integrase resistance mutations by Sanger sequencing. This, in turn, could have important clinical consequences because the documentation of minor integrase resistance mutations by NGS may be essential for managing the treatment switch to the long-acting cabotegravir plus rilpivirine injectable combination [18].

We expect most patients to achieve viral suppression with the global expansion of DTG-based antiretroviral therapy. This improvement will shift the clinical focus towards individual cases of incomplete virological suppression, like the one reported here, as well as similar cases in resource-limited countries. When this technology is available, we expect that NGS will also benefit patients from rural settings, who can only manage infrequent visits, by unraveling resistance mutations that may be missed by Sanger sequencing and evaluating cumulative drug adherence, as illustrated here. Thus, more work is warranted to characterize the emergence and maintenance vs. reversion dynamics of integrase resistance mutations against second-generation INSTIs and how NGS can be used as a diagnostic tool in clinical practice for optimal patient care.

### 3.5. Conclusions

We report a case of rapid fading of resistance mutations after patient-initiated treatment interruption that prevented their detection by Sanger sequencing despite high viral loads. Next-generation sequencing was superior and served as an indicator of cumulative drug adherence. We conclude that this method should be favored when managing patients with incomplete viral suppression.

## Figures and Tables

**Table 1:** Fold resistance against DTG and relative infectivity and fitness of H51Y, G118R, and H51Y+G118R viruses

Background	Genotype	FC (DTG)	Relative infectivity	Relative fitness
pNL4.3	WT	–	100%	100%
	H51Y	1.1	73%	100%
	G118R	7.2	38%	35%
	H51Y+G118R	28.3	43%	10%

DTG, dolutegravir; FC, fold change; WT, wild-type.
